# Supplementary material for: Digital eye strain syndrome among higher education health sciences students in Saudi Arabia: severity and preventive ergonomic practices
Source: PeerJ. 2024 Nov 14;12:e18423. doi: 10.7717/peerj.18423 (PMC11569780; doi:10.7717/peerj.18423)
Supplement: Supplemental Information 1 [file peerj-12-18423-s001.pdf]

متلازمة إجهاد العين الرقمي بين طلاب العلوم الصحية في التعليم العالي في المملكة العربية السعودية: شدتها والممارسات الارجونوميكس الوقائية.

**أ- الخصائص الاجتماعية**	
**1- العمر**..	
**2- النوع:** أ -ذكر ( )	ب-انثى ( )
**3- محل الاقامة: ....**	
**ب- التاريخ الطبي**	
**4- هل تعاني من أي مرض مزمن؟**	
أ- سكر ( )	ب- ضغط ( )
ت- اضطراب بالغدة الدرقية ( )	ث- أخر يذكر..............................
**5- هل لديك مشكلة في العين؟**	1-لا ( ) 2-نعم ( ) تذكر.............
**6- هل سبق لك إجراء عملية جراحية بالعين؟**	1-لا ( ) 2-نعم ( )
**7- هل ترتدي النظارات الطبية؟**	1-لا ( ) 2-نعم منذ عام او اقل ( ) 3. منذ أكثر من عام
**8- هل تتناول ادوية علاجية بصورة يومية؟**	-لا ( ) 2-نعم ( ) تذكر.............
**ج- بيانات استخدام الأجهزة الرقمية**	
**9- مدة استخدام الأجهزة الرقمية............ (سنة)**	
**10- أي من الأجهزة الرقمية التالية تستخدمه بشكل أكثر خلال يومك؟**<br>أ- الكمبيوتر المكتبي ( )<br>ب- الكمبيوتر المحمول (اللاب توب) ( )<br>ت- الجهاز اللوحي (التابلت) ( )<br>ث- الهاتف الذكي ( )	
**11- كم عدد ساعات استخدامك للأجهزة الرقمية يوميا تقريبا (بالعمل والمنزل)؟ ....(بالساعات)**	
**12- هل تستخدم نظارة حماية للعين أثناء استخدام الأجهزة الرقمية؟** 1-لا ( ) 2-نعم ( )	
**13- هل تستخدم عدسات لاصقة بالعين أثناء استخدام الأجهزة الرقمية؟** 1-لا ( ) 2-نعم ( )	
**14- ما هي مدة استخدام الشاشات الرقمية بدون استراحة (بالدقائق)؟..........................**	
**15- ما هي طريقة المذاكرة المفضلة لديك؟**	
- الأوراق المطبوعة غالبا	
- الشاشات الرقمية غالبا	
- كلاهما معا	
**16- كم عدد ساعات المحاضرات الافتراضية في الأسبوع؟**	
- أقل من ساعتين بالأسبوع	
- من 2 الى 9 ساعات بالأسبوع	
- 10 ساعات أو أكثر	

مقياس متلازمة اجهاد العين الرقمي

من فضلك حدد إلى أي مدى تعاني من هذه الأعراض (التكرار والحدة) خاصة أثناء أو بعد استخدام الأجهزة الرقمية لفترة طويلة خلال الشهر الماضي باختيار الرقم المناسب.

انتبه: إذا اخترت لا أعاني أبدا فلا تختار متوسطة أو شديدة الحدة

	التكرار			الحدة/الشدة	
**الأعراض**	**لا أعاني أبدا (0)**	**أعاني أحيانا (1)**	**أعاني بصورة متكررة (2)**	**متوسطة (1)**	**شديدة (2)**
أ- **أعراض متعلقة بالرؤية**					
1. زغللة بالعين (رؤية غير واضحة)					
2. رؤية مزدوجة					
3. صعوبة التركيز على الرؤية القريبة					
4. زيادة حساسية العين للضوء					
5. رؤية هالات ملونة حول الأشياء					
6. الشعور بأن البصر يقل أو يتدهور					
ب- **أعراض متعلقة بالعين**					
7. ألم بالعين					
8. جفون ثقيلة					
9. حرقان					
10. حكة					
11. احمرار					
12. جفاف					
13. زيادة إفراز الدموع					
14. كثرة الرمش أو رفة العين (غلق وفتح العين لا إراديا)					
15. الشعور بجسم غريب في العين					
16. الصداع					

مقياس تقييم مما راسات الأرجونوميكس للحماية من إجهاد العين الرقمي

اختر الإجابة المناسبة لممارساتك المتبعة للوقاية من إجهاد العين عند استخدام الشاشات الرقمية (حاسوب - جهاز لوحي- لاب توب – جوال) باختيار الرقم المناسب.

العبارات	أبدا	نادرا	أحيانا	غالبا	دائما
**وضعية الشاشة - Location of digital screen**					
1. هل تضع شاشة الجهاز تحت مستوى العين بحوالي 15-20 سم من منتصف الشاشة؟	1	2	3	4	5
2. هل تضع شاشة الجهاز على بعد 45-70 سم من العين؟	1	2	3	4	5
**إعدادات العرض على الشاشة - Display settings**					
3. هل تحافظ على درجة سطوع شاشة الجهاز منخفضة؟	1	2	3	4	5
4. هل تستخدم ألوان داكنة لخلفية الشاشة؟	1	2	3	4	5
5. هل تستخدم حجم مناسب للخط على الشاشة؟	1	2	3	4	5
**الإضاءة – Lightening**					
6. هل تضع الشاشة بطريقة مناسبة لتجنب الوهج أو الإضاءة الشديدة الصادرة من المصابيح أو النوافذ؟	1	2	3	4	5
7. هل تضع ستائر على النوافذ أثناء استخدام الشاشة الرقمية؟	1	2	3	4	5
8. هل تستخدم المصابيح الكهربائية ذات القوة الكهربائية المنخفضة (منخفضة الواط)؟	1	2	3	4	5
**حماية العين- Anti-glare screen or glasses/lenses**					
9. هل تستخدم شاشة واقية للحماية من وهج أو سطوع الشاشة؟	1	2	3	4	5
10. هل تستخدم نظارة أو عدسة لاصقة للعين أثناء استخدام الأجهزة الرقمية؟	1	2	3	4	5
**فترات الراحة - Rest breaks**					
11. هل تأخذ فترة راحة لعينيك لمدة 15 دقيقة كل ساعتين أثناء الاستخدام المستمر للجهاز؟	1	2	3	4	5
12. هل تمارس قاعدة العشرون دقيقة؟ (كل 20 دقيقة من وقت الشاشة، خذ استراحة لمدة 20 ثانية وركز على شيء على بعد 20 قدم أو ما يقارب 600 سم أو أكثر من نصف متر)	1	2	3	4	5
**الرمش أو رفة العين - Blinking**					
13. هل تمارس حركة رمش أو رفة العين (غلق وفتح العين) بشكل متكرر أثناء استخدام الجهاز؟	1	2	3	4	5
**حامل الوثيقة -Document holder**					
14. هل تستخدم حامل للوثيقة عند الكتابة على الجهاز أو تضع المستند فوق لوحة المفاتيح؟	1	2	3	4	5
**وضعية الجلوس -Seating position**					
15. هل تضع قدميك في وضع مريح أو على الأرض أثناء الجلوس على الجهاز؟	1	2	3	4	5
16. هل تقم بضبط أيدي الكرسي لسند الذراع ورسغ اليد أثناء الكتابة على لوحة المفاتيح؟	1	2	3	4	5
17. هل الكرسي مبطن بشكل مريح ويتوافق مع الجسم؟	1	2	3	4	5
