# Supplementary material for: Digital eye strain syndrome among higher education health sciences students in Saudi Arabia: severity and preventive ergonomic practices
Source: PeerJ. 2024 Nov 14;12:e18423. doi: 10.7717/peerj.18423 (PMC11569780; doi:10.7717/peerj.18423)
Supplement: Supplemental Information 2 [file peerj-12-18423-s002.pdf]

Digital Eye Strain Syndrome Among Higher Education Health Sciences Students in Saudi Arabia: Severity and Preventive Ergonomic Practices.

**Sociodemographic data**
**1. Age…………….**
**2. Gender**
- Male
- Female
**3. Residence**
- Riyadh
- Jeddah
- Dammam
**Medical history**
**4. Do you have a chronic disease?**
- Diabetes
- Hypertension
- Thyroid gland problems
- Others …………..
**5. Do you have an eye problem?**
- No
- Yes
- If yes, mention it…………………
**6. Do you have a history of eye surgery?**
- No
- Yes
**7. Do you wear glasses?**
- No
- Yes
**8. Do you use medication daily?**
- No
- Yes
- If yes, mention it…………………
**Digital devices use data**
**9. Duration of digital device use in years…………….**
**10. Which of the following devices are used more daily?**
- Desktop computer
- Laptop
- Tablet
- Smartphone
**11. How many average daily hours are spent in digital device use?.......................**
**12. Do you use protective eyeglasses during digital device use?**
- No
- Yes
**13. Do you use eye lenses during digital device use?**
- No
- Yes
**14. What is the duration of screen use without a break (in minutes)?...............**

**15. What is your preferred method of studying?**
- Mainly printed paper
- Mainly digital screen
- Both are equally used
**16. How many virtual classes hours per week?**
- Less than 2 hours
- From 2 to 9 hours
- 10 or more hours

Digital eye strain syndrome questionnaire

By choosing the appropriate number, please indicate the extent to which you experience these symptoms (frequency and severity), especially during or after using a digital device for a long period during the past month.

Don't forget: If you choose "Never" in the frequency section, don't select medium or severe in the severity section.

**Symptoms**	**Severity**		**Frequency**		
	**Severe (2)**	**Medium (1)**	**Often or always (2)**	**Occasional (1)**	**Never (0)**
**Vision-related symptoms**					
1. Blurred vision					
2. Double vision					
3. Difficulty focusing for near vision					
4. Increased sensitivity to light					
5. Colored halos around objects					
6. Feeling that eyesight is worsening					
**Eye related symptoms**					
7. Eye pain					
8. Heavy eyelids					
9. Burning					
10. Itching					
11. Redness					
12. Dryness					
13. Tearing					
14. Excessive blinking					
15. A feeling of a foreign body in the eyes					
16. Headache					

<u>Preventive ergonomic practices scale</u>

Choose the appropriate answer for your preventive ergonomic practices against eye strain when using digital screens (computer - tablet - laptop - mobile) by choosing the appropriate number.

	Never	Rarely	Sometimes	Often	Always
**Location of digital screen**					
1. Do you place the device screen approximately 15-20 cm below eye level from the center of the screen?	**1**	**2**	**3**	**4**	**5**
2. Do you place the device screen 45-70 cm from the eye?	**1**	**2**	**3**	**4**	**5**
**Display settings**					
3. Do you keep your device's screen brightness low?	**1**	**2**	**3**	**4**	**5**
4. Do you use dark colors for your screen background?	**1**	**2**	**3**	**4**	**5**
5. Are you using the appropriate font size on the screen?	**1**	**2**	**3**	**4**	**5**
**Lightening**					
6. Are you positioning the screen appropriately to avoid glare or harsh light from lamps or windows?	**1**	**2**	**3**	**4**	**5**
7. Do you put curtains on your windows while using a digital screen?	**1**	**2**	**3**	**4**	**5**
8. Are you using low-wattage light bulbs?	**1**	**2**	**3**	**4**	**5**
**Anti-glare screen or glasses/lenses**					
9. Do you use a protective screen against screen glare?	**1**	**2**	**3**	**4**	**5**
10. Do you use glasses or contact lenses while using digital devices?	**1**	**2**	**3**	**4**	**5**
**Rest breaks**					
11. Do you take a 15-minute break every two hours during digital device use?	**1**	**2**	**3**	**4**	**5**
12. Do you practice the 20-20-20 rule? (Every 20 minutes of screen time, take a 20-second break and focus on something 20 feet away or more than half a meter away.)	**1**	**2**	**3**	**4**	**5**
**Blinking**					
13. Do you practice frequent blinking while using the digital device?	**1**	**2**	**3**	**4**	**5**
**Document holder**					
14. Do you use a document holder when typing on the device or place the document on the keyboard?	**1**	**2**	**3**	**4**	**5**

Seating position					
15. Do you place your feet in a comfortable position or on the floor while using the digital device?	**1**	**2**	**3**	**4**	**5**
16. Do you adjust the chair's handles to support the arm and wrist while typing on the keyboard?	**1**	**2**	**3**	**4**	**5**
17. Is the chair comfortably padded and conforms to the body?	**1**	**2**	**3**	**4**	**5**
